# Machine learning differentiation of rheumatoid arthritis-Sjögren’s syndrome overlap from Sjögren’s syndrome with polyarthritis

**DOI:** 10.3389/fimmu.2025.1614631

**Published:** 2025-07-08

**Authors:** Minzhi Gan, Yong Peng, Ying Ying, Keyue Zhang, Yong Chen

**Affiliations:** Department of Rheumatology and Immunology, Ningbo NO.2 Hospital, Ningbo, Zhejiang, China

**Keywords:** rheumatoid arthritis, Sjögren’s syndrome, machine learning, random forest, differential diagnosis, autoimmune diseases, arthritis

## Abstract

**Objective:**

This study aimed to evaluate the utility of machine learning algorithms in differentiating rheumatoid arthritis-Sjögren’s syndrome overlap (RA-SS) from Sjögren’s syndrome with polyarthritis (SS-PA), and to identify key factors influencing diagnostic differentiation.

**Methods:**

This retrospective analysis included 106 RA-SS and 135 SS-PA patients randomized 7:3 into training and validation sets. Clinical, laboratory, and radiographic data were collected. Least Absolute Shrinkage and Selection Operator (LASSO) regression facilitated feature selection before constructing diagnostic models using four machine learning algorithms, with feature importance quantified through SHapley Additive exPlanations (SHAP).

**Results:**

The random forest algorithm demonstrated superior performance (AUC=0.854, 95% CI: 0.747-0.944) compared to other machine learning algorithms. SHAP analysis identified anti-CCP level, rheumatoid factor (RF) level, erosive joint count, anti-SSA/Ro60 antibodies, and C-reactive protein (CRP) as critical discriminating factors between RA-SS and SS-PA.

**Conclusion:**

The random forest algorithm demonstrates promising clinical potential for RA-SS and SS-PA differential diagnosis, with diagnostic efficiency surpassing traditional logistic regression (LR), offering a new approach for clinical differentiation.

## Introduction

Sjögren’s syndrome (SS) is a chronic autoimmune disorder characterized by lymphocytic infiltration of exocrine glands, resulting primarily in xerophthalmia and xerostomia. In addition to glandular involvement, SS can affect multiple organ systems including joints, nervous system, lungs, and kidneys (1–3). Evidence from observational studies indicates that arthritic symptoms occur in 30-60% of SS patients (4). A longitudinal study following 521 patients documented that 44% developed extra-glandular manifestations, including arthritis and neurological involvement, within six years of initial sicca symptoms. Joint manifestations typically present as polyarticular pain and swelling, predominantly affecting small joints of the hands, wrists, and feet (5). Although most patients exhibit non-erosive arthritis, a subset of primary SS patients develop destructive joint changes clinically resembling rheumatoid arthritis (RA) (6). SS may occur as a primary disorder or in association with other connective tissue diseases, with RA representing one of the most frequent comorbidities.

RA is an autoimmune disease characterized by synovial inflammation and hyperplasia, resulting in cartilage degradation and bone deformity. Cross-sectional analyses demonstrate significantly higher prevalence of joint tenderness, swelling, and pain in patients with RA-SS overlap (7). Currently, no pathognomonic laboratory tests exist for definitively diagnosing either RA or SS. Both diagnoses require comprehensive assessment of symptoms, clinical signs, and laboratory parameters. SS patients with polyarthritis (SS-PA) may manifest joint distribution patterns clinically indistinguishable from RA-SS, thereby confounding differential diagnostic assessment.

Furthermore, rheumatoid factor (RF) remains a widely utilized diagnostic marker due to its accessibility, standardization across laboratories, and historical significance in classification criteria (8, 9). Clinical data indicate that approximately 50% of early RA patients may be RF-negative, while 50-80% of primary SS patients may test RF-positive (10). Anti-cyclic citrullinated peptide antibody (anti-CCP) exhibits superior specificity for RA diagnosis. Despite this advantage, recent studies identified anti-CCP positivity in 7.5-10% of SS patients (11). These overlapping serological profiles, combined with the absence of definitive radiographic changes in early disease stages, create differential diagnostic uncertainty that can delay appropriate therapeutic intervention. Accurate differentiation between these conditions is essential for therapeutic decision-making, as RA-SS patients require earlier and more aggressive disease-modifying antirheumatic drug therapy to prevent irreversible joint damage.

Previous investigations frequently utilized conventional statistical approaches such as logistic regression (LR) to analyze multiple variables and evaluate diagnostic performance through receiver operating characteristic (ROC) curves (12–14). Despite their capacity for multivariate analysis, these methodologies exhibit inherent constraints in dimensionality reduction, feature selection, and non-linear pattern recognition, particularly when confronted with complex interparametric relationships across clinical, serological, and radiographic domains.

Recently, artificial intelligence has demonstrated remarkable progress in autoimmune disease diagnostics, with recent comprehensive reviews highlighting its transformative potential across rheumatological applications (15). Machine learning approaches have shown substantial advantages in handling heterogeneous clinical datasets, with the random forest algorithm integrating hand imaging and functional metrics demonstrating exceptional discriminatory capacity between RA patients and healthy controls (16). More complex machine learning methods, particularly deep learning approaches, have been increasingly applied to rheumatological imaging tasks. Recent studies have demonstrated the effectiveness of deep learning radiomics fusion models for ultrasound-based bone erosion identification in RA patients, achieving area-under-curve values exceeding 0.93 in external validation (17). Deep learning approaches applied to metacarpophalangeal joint ultrasound imaging in RA have shown excellent discriminative ability between normal and pathological synovium, with performance metrics exceeding 0.8 in AUC (18). These diverse machine learning applications demonstrate the potential to identify complex feature patterns undetectable by conventional statistical methods. Accordingly, this study aims to develop machine learning-based differential diagnostic models incorporating demographic characteristics, clinical manifestations, laboratory parameters, and radiographic findings to establish robust differential diagnostic algorithms distinguishing SS-PA from RA-SS, thereby providing more precise diagnostic criteria for clinical implementation.

## Materials and methods

This study was approved by the Ethics Committee of Ningbo No.2 Hospital (YJ-NBEY-KY-2023-143-01) and adhered to all principles established in the Declaration of Helsinki. Given the retrospective, observational design, the ethics committee granted a waiver of individual informed consent. Patient confidentiality was maintained through comprehensive deidentification procedures, with systematic removal of all personal identifiers from electronic health records prior to analysis in accordance with institutional privacy standards.

### Study population

Clinical data from arthritis patients presenting to the Department of Rheumatology and Immunology between January 2018 and June 2024 were retrospectively analyzed. RA was diagnosed according to the 2010 Rheumatoid Arthritis Classification Criteria (19). SS was defined according to the 2002 American-European Consensus Group (AECG) or 2012 American College of Rheumatology (ACR) classification criteria (20, 21). All patients underwent standardized diagnostic and treatment protocols administered by experienced rheumatology specialists.For this study, RA-SS patients were defined as those concurrently meeting both RA and SS criteria. Patients were included if they had complete demographic and laboratory data and had undergone standard imaging during their initial consultation. Exclusion criteria comprised severe hepatic or renal impairment, concomitant malignancy, other rheumatic autoimmune diseases (ankylosing spondylitis, gout, systemic lupus erythematosus), the presence of trauma, infection, or other bone and joint disorders.

**Figure 1:**
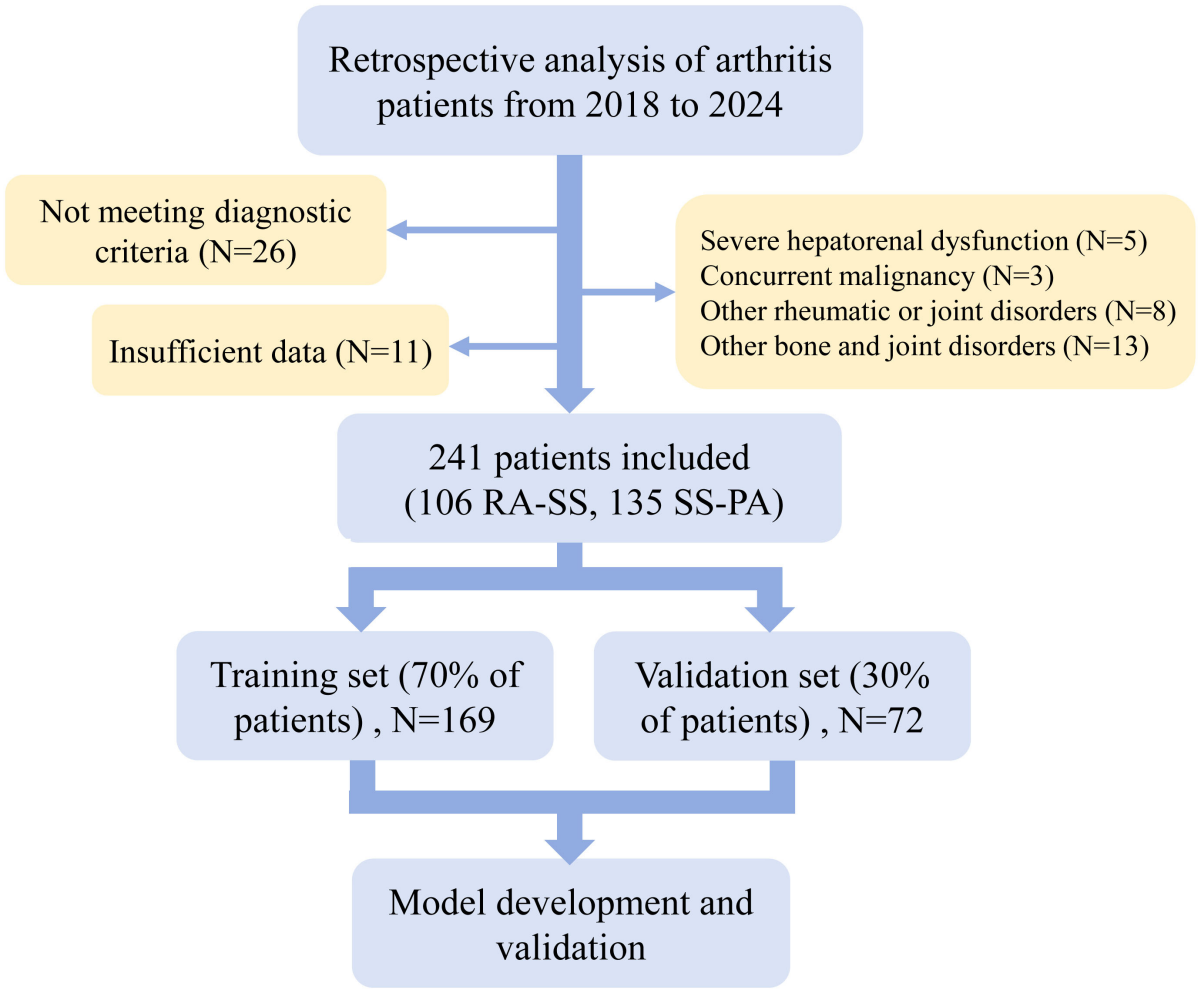
Flow diagram of patient selection for this study. RA-SS, rheumatoid arthritis-Sjögren’s syndrome overlap; SS-PA, Sjögren’s syndrome with polyarthritis.

Ultimately, a total of 241 patients met the selection criteria, including 106 with RA-SS and 135 with SS-PA. Patients were randomized to training and validation cohorts (7:3 ratio), resulting in 169 training set (74 RA-SS, 95 SS-PA) and 72 validation set (32 RA-SS, 40 SS-PA), as illustrated in **Figure 1**.

### Radiological examination

Radiological assessment employed peripheral joint radiographs as the primary imaging modality, with particular emphasis on bilateral hands and wrists. Magnetic resonance imaging was selectively performed in cases requiring further clarification of bone erosion. Imaging analysis documented structural abnormalities including erosive changes, symmetrical joint involvement patterns, and distribution of affected joint sites. All radiological data underwent independent evaluation by two experienced radiologists, with discrepancies resolved through consensus discussion.

### Data collection

Data acquisition comprised demographic variables (age, sex, disease duration), primary clinical manifestations (sicca symptoms, cutaneous lesions, parotid enlargement, Raynaud’s phenomenon, tender, and swollen joint counts), and systemic organ involvement (hematological, neurological, pulmonary, and renal manifestations). Laboratory testing targeted hematological indices, inflammatory markers, autoantibody profiles, and immunological parameters.

All laboratory examinations adhered to standardized protocols. Complete blood count and erythrocyte sedimentation rate (ESR) were measured using a Beckman Coulter LH 750 analyzer (Beckman Coulter Inc., California, USA). Additionally, antinuclear antibodies (ANA) were evaluated by indirect immunofluorescence (positive: titer ≥1:80), while anti-Ro/SSA and anti-La/SSB antibodies were detected via immunoblotting. Immunonephelometry measured rheumatoid factor (positive: ≥20 IU/mL), complement component 3 (C3) and 4 (C4), C-reactive protein (CRP), and immunoglobulins. Anti-cyclic citrullinated peptide (anti-CCP) antibodies were quantified using chemiluminescence immunoassay (positive: >5 U/mL).

### Data processing

The analytical framework incorporated 46 feature variables encompassing demographic characteristics, clinical manifestations, radiological findings, and laboratory parameters. Original medical records were reviewed by two investigators independently to ensure data extraction accuracy. Continuous variables underwent z-score standardization (mean=0, standard deviation=1), while categorical variables were transformed into numerical features through one-hot encoding. Least Absolute Shrinkage and Selection Operator (LASSO) regression with 10-fold cross-validation was implemented for feature selection. The optimal regularization parameter λ was determined through cross-validation on the training dataset. The final diagnostic model included variables that retained non-zero coefficients with stable magnitudes across multiple iterations of the cross-validation procedure.

### Model development

Four machine learning algorithms were developed for SS-PA and RA-SS differentiation: LR, support vector machine (SVM), random forest, and extreme gradient boosting (XGBoost). LR incorporated L2 regularization, with strength parameters logarithmically spaced from 0.001 to 10. The SVM algorithm utilized a radial basis function kernel, systematically evaluating kernel parameter γ (logarithmically spaced from 0.001 to 0.1) and penalty coefficient C (logarithmically spaced from 1 to 100), with a tolerance of 0.0001. Random forest parameters were optimized across tree numbers (100-500), maximum depth (4-8), and minimum samples for split (2-10), employing bootstrap sampling with a ratio of 0.8. For XGBoost, learning rates (0.01-0.2), number of trees (100-300), and maximum depth (3-6) were evaluated, with subsample and feature sample ratios fixed at 0.8. Early stopping criteria monitored validation loss. Grid search optimization determined optimal parameter combinations through iterative evaluation across the cross-validation folds.

Model performance was assessed using 10-fold cross-validation. The training set underwent random division into 10 subsets, with models iteratively trained on nine subsets and validated on the remaining subset. The complete model training code is provided in the **Supplementary Appendix**.

### Statistical analysis

Continuous variables were expressed as mean (SD) or median (Q1, Q3) based on their distribution normality, while categorical variables were presented as numbers (percentages). Comparative analyses between groups were conducted using independent t-test or Wilcoxon rank-sum test for continuous variables and chi-square test for categorical variables. Model performance was evaluated using accuracy, sensitivity, specificity, area under the receiver operating characteristic curve (AUC), and additional diagnostic metrics, with 95% confidence intervals calculated through bootstrap resampling. The DeLong test was employed to compare AUCs between different models. Subsequently, the SHapley Additive exPlanations (SHAP) method was utilized to quantify feature contributions to model classification and rank overall feature importance.

Data preprocessing and model development were implemented using Python (version 3.8) and scikit-learn (version 1.0) library. SHAP analysis was performed using the SHAP library (version 0.40). Statistical significance was set at P < 0.05.

## Results

### Baseline characteristics

The study enrolled 241 patients, predominantly female (95.02%), with median age of 54 years and median disease duration of 60 months. Exocrine glandular dysfunction presented as ocular and oral dryness in 81.33% and 69.29% of patients, respectively. Articular involvement was prevalent, characterized by symmetrical distribution (86.72%) and hand joint arthritis (77.18%), with clinical features of synovitis and radiographic erosions in a subset of patients. Extraglandular manifestations included Raynaud’s phenomenon (34.02%), lung (19.09%) and neurological (20.75%) involvement, and renal impairment (14.94%). No significant differences in demographic or clinical parameters were observed between training and validation sets (**Table 1**, all P>0.05).

**Table 1:** Comparison of demographic and clinical features between training and validation sets.

Factors	All (n=241)	Training set (n=169)	Validation set (n=72)	P value
Age, years	54 (42, 69)	54 (41, 68)	56.5 (43.75, 72)	0.365
Female, N (%)	229 (95.02)	160 (94.67)	69 (95.83)	0.956
Diabetes duration, months	60 (29, 82)	59 (29, 83)	61 (28.25, 82)	0.820
Clinical manifestations				
Xerophthalmia, N (%)	196 (81.33)	136 (80.47)	60 (83.33)	0.602
Xerostomia, N (%)	167 (69.29)	119 (70.41)	48 (66.67)	0.564
Salivary gland enlargement, N (%)	29 (12.03)	21 (12.43)	8 (11.11)	0.774
Rash, N (%)	15 (6.22)	11 (6.51)	4 (5.56)	0.991
Raynaud phenomenon, N (%)	82 (34.02)	57 (33.73)	25 (34.72)	0.881
Symmetric arthritis, N (%)	209 (86.72)	146 (86.39)	63 (87.50)	0.816
Hand arthritis, N (%)	186 (77.18)	132 (78.11)	54 (75.00)	0.599
Swollen joint count	7 (6, 9)	8 (6, 9)	7 (6, 9)	0.545
Tender joint count	9 (7, 11)	9 (7, 11)	9 (7, 11)	0.702
Erosive joint count	2 (1, 4)	2 (1, 5)	2 (1, 4)	0.893
Anemia, N (%)	57 (23.65)	39 (23.08)	18 (25)	0.748
Leukopenia, N (%)	23 (9.54)	16 (9.47)	7 (9.72)	0.951
Lymphopenia, N (%)	26 (10.79)	19 (11.24)	7 (9.72)	0.728
Thrombocytopenia, N (%)	16 (6.64)	12 (7.10)	4 (5.56)	0.874
Lung involvement, N (%)	46 (19.09)	32 (18.93)	14 (19.44)	0.927
Renal involvement, N (%)	36 (14.94)	25 (14.79)	11 (15.28)	0.923
Nervous system involvement, N (%)	50 (20.75)	36 (21.30)	14 (19.44)	0.745

### Laboratory evaluation

Laboratory results demonstrated significant immunological abnormalities in the study population (**Table 2**). Autoantibody profiles showed high positivity rates for ANA (78.84%), anti-SSA/Ro52 (70.12%), anti-SSA/Ro60 (59.75%), RF (61.41%), and anti-CCP (47.30%). Immunological parameters showed a median IgG level of 16.75 g/L, and hypergammaglobulinemia was present in 40.66% of patients. All laboratory parameters, including hematological indices, inflammatory markers, autoantibody profiles, and immunological parameters showed comparable distributions between training and validation sets (all P>0.05).

**Table 2:** Comparison of laboratory parameters between training and validation sets.

Factors	All (n=241)	Training set (n=169)	Validation set (n=72)	P value
PLT, ×10^9^/L	236 (177, 279)	236 (180, 282)	236 (173, 277)	0.658
WBC, ×10^9^/L	6.60 (5.24, 7.92)	6.55 (5.11, 7.70)	6.70 (5.37, 8.32)	0.244
ANC, ×10^9^/L	3.70 (2.73, 4.75)	3.73 (2.80, 4.75)	3.56 (2.56, 4.72)	0.494
ALC, ×10^9^/L	2.64 (1.51, 3.72)	2.61 (1.51, 3.69)	2.74 (1.60, 3.86)	0.499
Hb, g/L	119 (114, 125)	118 (114, 125)	119 (114, 125)	0.636
Alb, g/L	40.11 (37.69, 44.81)	40.04 (37.63, 44.61)	40.13 (37.81, 45.29)	0.492
Glb, g/L	32.25 (26.02, 34.41)	32.23 (25.78, 34.46)	32.30 (26.17, 34.40)	0.767
Cr, μmol/L	59.85 (49.45, 79.90)	59.85 (49.45, 79.90)	61.52 (49.58, 79.97)	0.906
BUN, mmol/L	5.54 (4.33, 6.25)	5.56 (4.45, 6.25)	5.52 (4.19, 6.13)	0.464
CRP, mg/L	4.33 (2.73, 5.97)	4.30 (2.81, 6.10)	4.36 (2.63, 5.84)	0.814
ESR, mm/h	18 (13, 22)	18 (12, 23)	17 (13, 22)	0.531
ANA, 1/titer	210 (120, 300)	210 (120, 290)	210 (127.5, 302.5)	0.410
ANA positivity, N (%)	190 (78.84)	133(78.70)	57 (79.17)	0.935
Anti-SSA/Ro52 positivity, N (%)	169 (70.12)	118 (69.82)	51 (70.83)	0.875
Anti-SSA/Ro60 positivity, N (%)	144 (59.75)	102 (60.36)	42 (58.33)	0.770
Anti-SSB/La positivity, N (%)	58 (24.07)	42 (24.85)	16 (22.22)	0.662
Anti-dsDNA positivity, N (%)	19 (7.88)	13 (7.69)	6 (8.33)	0.866
Anti-Sm positivity, N (%)	14 (5.81)	10 (5.92)	4 (5.56)	0.849
ACA positivity, N (%)	16 (6.64)	11 (6.51)	5 (6.94)	0.874
C3, g/L	0.92 (0.69, 1.07)	0.94 (0.63, 1.08)	0.93 (0.70, 1.08)	0.568
C4, g/L	0.23 (0.17, 0.29)	0.23 (0.17, 0.29)	0.24 (0.17, 0.30)	0.426
RF positivity, N (%)	148 (61.41)	105 (62.13)	43 (59.72)	0.725
RF, IU/mL	41.20 (12.91, 72.84)	41.20 (12.52, 72.84)	40.87 (13.64, 75.57)	0.882
Ig G, g/L	16.75 (14.23, 21.37)	16.62 (14.25, 20.32)	16.84 (14.10, 21.32)	0.573
Ig A, g/L	2.85 (2.10, 4.32)	2.71 (2.08, 4.31)	2.87 (2.28, 4.29)	0.589
Ig M, g/L	1.18 (0.84, 1.75)	1.14 (0.84, 1.65)	1.18 (0.87, 1.75)	0.781
Anti-CCP, U/mL	4.97 (2.21, 20.88)	4.95 (2.23, 20.85)	5.04 (2.23, 21.55)	0.610
Anti-CCP positivity	114 (47.30)	80 (47.34)	34 (47.22)	0.987
HGG, N (%)	98 (40.66)	68 (40.24)	30 (41.67)	0.836

PLT, platelet count; WBC, white blood cell count; ANC, absolute neutrophil count; ALC, absolute lymphocyte count; Hb, hemoglobin; Alb, albumin; Glb, globulin; Cr, creatinine; BUN, blood urea nitrogen; CRP, C-reactive protein; ESR, erythrocyte sedimentation rate; ANA, antinuclear antibody; Anti-SSA/Ro52, anti-sjögren’s syndrome-related antigen A/Ro52 antibody; Anti-SSA/Ro60, anti-sjögren’s syndrome-related antigen A/Ro60 antibody; Anti-SSB/La, anti-sjögren’s syndrome-related antigen B/La antibody; Anti-dsDNA, anti-double-stranded DNA antibody; Anti-Sm, anti-smith antibody; ACA, anti-centromere antibody; C3, complement component 3; C4, complement component 4; RF, rheumatoid factor; IgG, immunoglobulin G; IgA, immunoglobulin A; IgM, immunoglobulin M; Anti-CCP, anti-cyclic citrullinated peptide antibody; HGG, hypergammaglobulinemia.

### Feature selection

**Figure 2:**
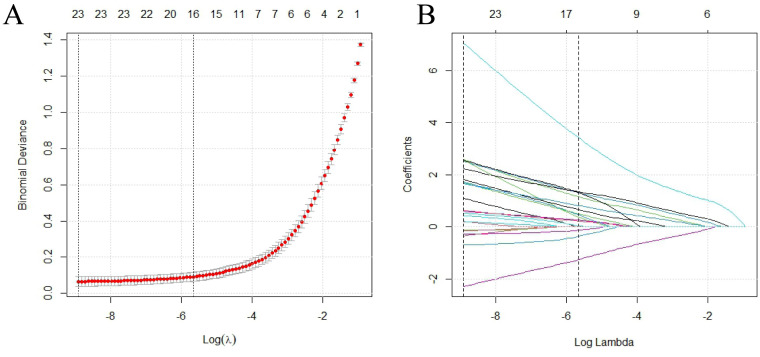
LASSO regression analysis for feature selection. **(A)** Cross-validated deviance curve against log(λ), where λ is the regularization parameter; vertical dotted lines indicate optimal λ values; **(B)** LASSO coefficient profiles across log(λ) values. LASSO, least absolute shrinkage and selection operator.

LASSO regression identified 18 statistically significant factors from the initial 46 features through 10-fold cross-validation for optimal penalty parameter determination (**Figure 2A**). The selected feature set encompassed variables across multiple dimensions, including clinical symptomatology, immunological parameters, and radiographic findings, integrating key indicators of joint involvement and immunological abnormalities. Coefficient trajectory analysis (**Figure 2B**) revealed notable stability of autoantibody markers throughout the penalization process, with anti-CCP antibodies and RF maintaining relatively high coefficient values. In addition, bone erosion-related parameters exhibited consistent contributions across most penalization levels.

### Model evaluation

**Figure 3:**
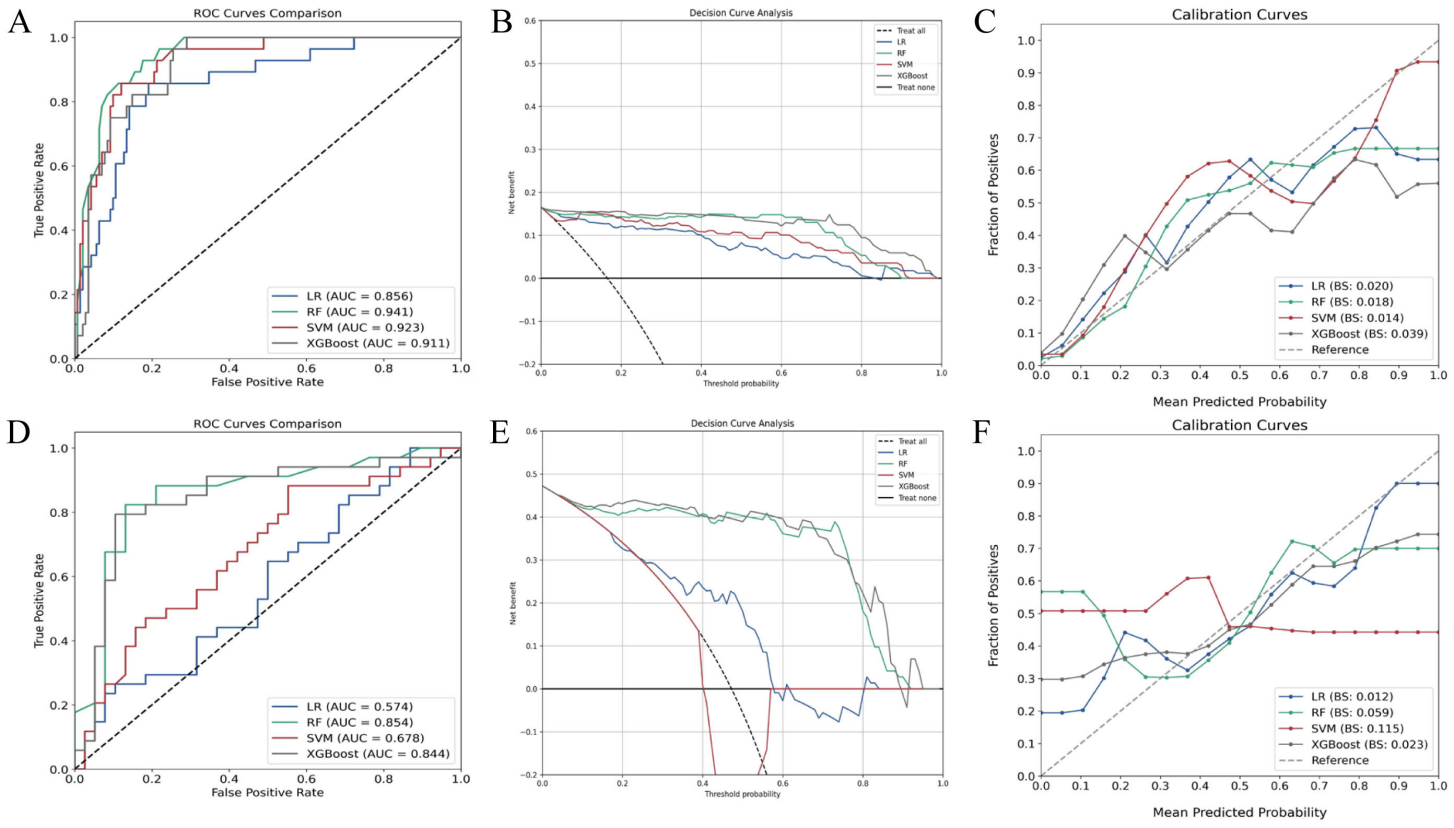
Performance evaluation of various machine learning classification algorithms. **(A)** ROC curves of algorithms in the training set; **(B)** Decision curve analysis in the training set; **(C)** Calibration curves in the training set; **(D)** ROC curves in the validation set; **(E)** Decision curve analysis in the validation set; **(F)** Calibration curves in the validation set. RF, random forest; SVM, support vector machine; XGBoost, extreme gradient boosting; LR, logistic regression; ROC, receiver operating characteristic.

**Figure 4:**
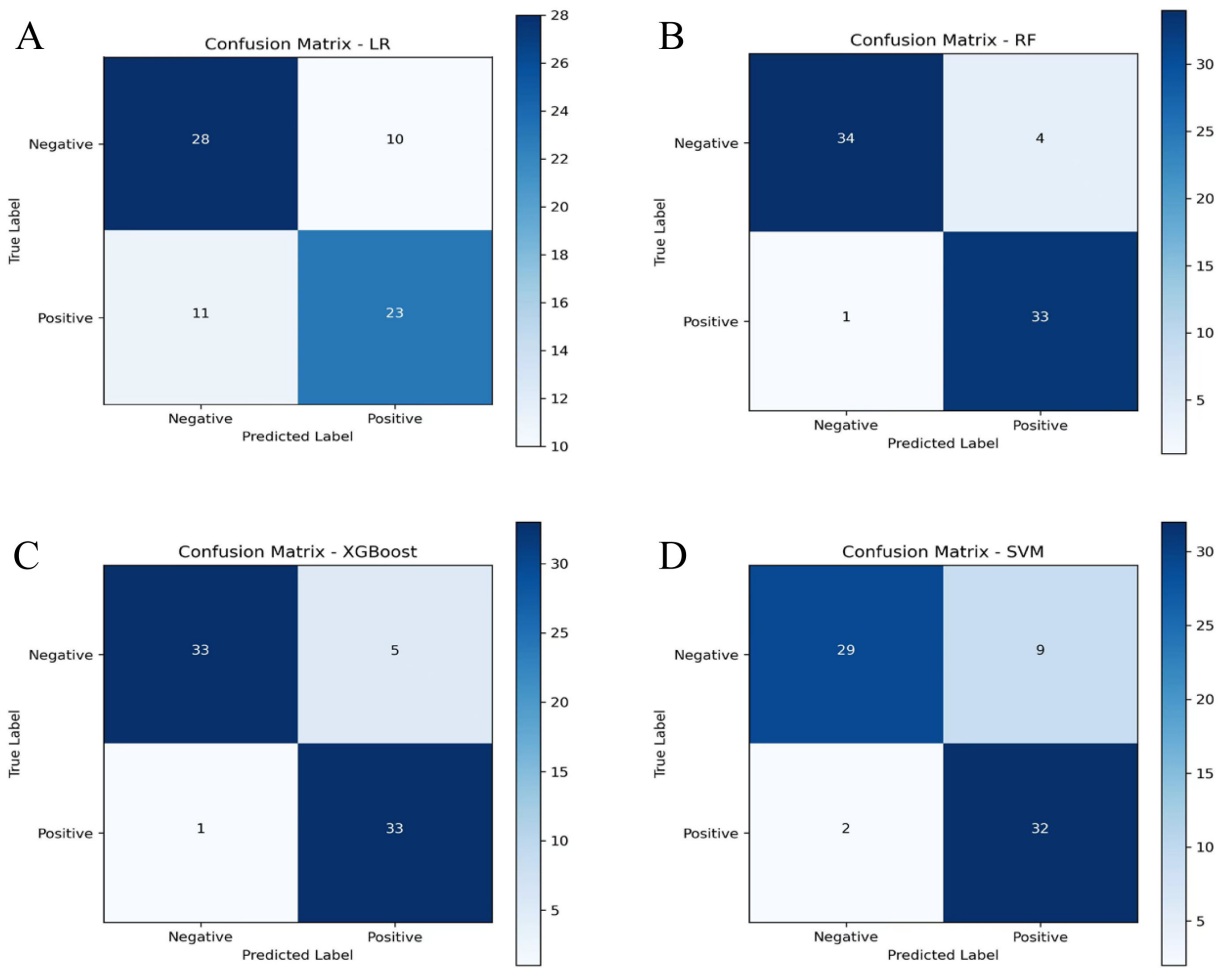
Confusion matrices of machine learning algorithms in the validation set display the distribution of true and predicted classifications. **(A)** Confusion matrix of the LR algorithm; **(B)** Confusion matrix of the RF algorithm; **(C)** Confusion matrix of the XGBoost algorithm; **(D)** Confusion matrix of the SVM algorithm. RF, random forest; SVM, support vector machine; XGBoost, extreme gradient boosting; LR, logistic regression.

In the training set, all algorithms (random forest, SVM, XGBoost, LR) exhibited excellent discriminative capacity with AUC values exceeding 0.85, with random forest demonstrating superior overall performance (**Figures 3A–C**). Evaluation on the validation set revealed significant inter-algorithm performance differences. As illustrated in **Figure 3D**, random forest and XGBoost maintained robust discriminative capacity, with AUC values of 0.854 and 0.844, respectively. In contrast, SVM and LR showed marked performance deterioration, with AUC values of 0.678 and 0.574, respectively. Statistical comparison by the DeLong test confirmed significant superiority of random forest and XGBoost over SVM and LR (p<0.05). Decision curve analysis (**Figure 3E**) demonstrated sustained high net benefit for random forest and XGBoost across the 0.4-0.8 threshold probability range, while SVM exhibited substantial decline beyond 0.4 threshold and LR consistently yielded suboptimal net benefit. Calibration curves (**Figure 3F**) revealed comparable probability estimation properties between random forest and XGBoost algorithms. Comprehensive evaluation of performance metrics showed identical sensitivity between random forest and XGBoost. However, random forest demonstrated modest superiority in accuracy, specificity, precision, F1 score, and kappa coefficient (**Figure 4**; **Table 3**).

**Table 3:** Comparative model performance metrics in the validation set.

Metrics	Random forest	XGBoost	SVM	LR
AUC (95% CI)	0.854 (0.747-0.944)	0.844 (0.738-0.939)	0.678 (0.551-0.808)*	0.574 (0.446-0.704)*
Accuracy	0.83	0.82	0.64	0.56
Sensitivity	0.79	0.79	0.38	0.26
Specificity	0.87	0.84	0.87	0.82
Precision	0.84	0.82	0.72	0.56
Recall	0.79	0.79	0.38	0.26
F1	0.82	0.81	0.50	0.36
Kappa	0.66	0.64	0.26	0.08
Brier	0.14	0.15	0.23	0.25

As against the random forest, *p < 0.05. SVM, support vector machine; XGBoost, extreme gradient boosting; LR, logistic regression; AUC, area under the curve.

### Feature importance

**Figure 5:**
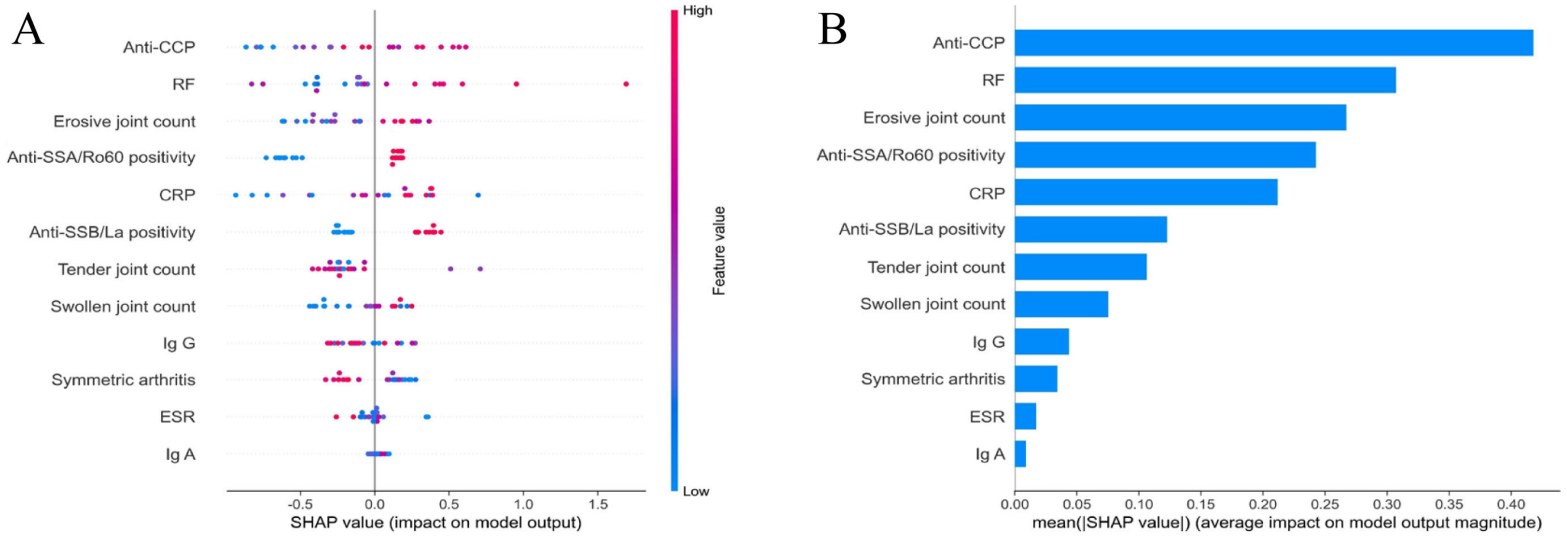
SHAP analysis of feature importance in the random forest algorithm. **(A)** Distribution of SHAP values for each feature, demonstrating the impact of individual predictors on model output. Each point represents a single observation, with color indicating feature value; **(B)** Mean absolute SHAP values quantifying the average magnitude of feature contributions to model outputs, arranged in descending order of importance. SHAP, SHapley Additive exPlanations.

The SHAP value analysis revealed key features influencing model output (**Figure 5A**). Anti-CCP level emerged as the most discriminative factor, with elevated values positively correlating with RA-SS classification. Moreover, RF demonstrated a positive association effect, with increased levels enhancing RA-SS identification probability. Erosive joint count, anti-SSA/Ro60 positivity, and CRP level exhibited positive associations with RA-SS classification.

Mean absolute SHAP value analysis (**Figure 5B**) further quantified the impact magnitude of each feature, with anti-CCP level demonstrating the highest mean absolute SHAP value (approximately 0.4), substantially exceeding other indicators. RF level, erosive joint count, anti-SSA/Ro60 positivity, and CRP level displayed moderate contributions. Comparatively, anti-SSB/La, tender joint count, swollen joint count, IgG, symmetric arthritis, ESR, and IgA exhibited relatively limited influence in distinguishing between the two disease categories.

## Discussion

RA-SS represents an autoimmune pathological overlap between RA and SS with complex and severe clinical manifestations. According to large-scale data from the American Corrona RA registry, the comorbidity rate of SS in RA patients reaches approximately 30% (22). SS coexistence likely exacerbates RA disease burden and diminishing quality of life. It also correlates with increased disease severity, elevated lymphoma risk, and poorer prognosis (23). Joint structural damage constitutes a critical prognostic factor in RA-SS patients, manifesting as progressive bone erosion, joint space narrowing, and osseous destruction. Non-erosive arthritis, conversely, suggests SS-PA consideration (24). Based on this clinical distinction, early identification and intervention for RA-SS hold significant clinical importance for preventing irreversible joint structural damage, potentially enhancing patients’ long-term quality of life. This investigation employed machine learning algorithms to construct diagnostic models, providing innovative methodology for early RA-SS identification. Results demonstrated that among four evaluated machine learning models, the random forest algorithm exhibited superior performance with validation set AUC reaching 0.854, surpassing the traditional LR algorithm. SHAP analysis identified anti-CCP level as the most discriminative factor, and RF level, erosive joint count, anti-SSA/Ro60 positivity, and CRP level showed close associations with RA-SS diagnosis. Although these factors represent established biomarkers, our integration of joint manifestations with serological parameters through machine learning methodology enhanced differential diagnostic precision and provided objectively quantified diagnostic parameters for clinical practice.

This study population exhibited a predominantly female gender distribution with peak incidence in middle-aged adults, which is a demographic pattern consistent with established literature on SS (25, 26). Sicca symptoms (xerostomia and xerophthalmia) were prevalent among subjects and demonstrated similar patterns across different disease subtypes, reflecting the exocrine gland dysfunction characteristic of SS in both patient groups. Regarding treatment approaches, RA-SS patients have historically required more intensive immunosuppressive regimens. Observational studies demonstrated higher proportions of glucocorticoid and methotrexate utilization in RA-SS patients compared to SS-PA counterparts, correlating with increased disease activity and erosive joint inflammation (27). Both patient groups typically receive DMARD therapy, though specific medication selection and dosing may differ based on predominant disease manifestations. This therapeutic distinction further underscores the importance of precise differential diagnosis in guiding clinical decision-making and optimizing treatment outcomes.

Analysis of joint involvement patterns reveals distinct disease-specific manifestations. This study employed comprehensive articular assessment metrics including symmetric arthritis proportion, hand joint involvement proportion, and quantification of swollen, tender, and erosive joints, that collectively reflect distribution patterns, inflammatory burden, and structural integrity. Patients with RA-SS demonstrate persistent symmetric polyarthropathy with characteristic hand joint involvement, sustained synovitis, and distinctive marginal bone erosions on radiographic evaluation (28, 29). Primary SS manifests articular involvement as its predominant extraglandular feature, typically characterized by intermittent presentation and preservation of structural integrity on imaging studies (30). The clinical distinction becomes particularly challenging in early-stage or atypical presentations, where significant phenotypic overlap exists between SS-PA and RA-SS. Mohammed et al. (31) reported that 53% of SS-PA patients exhibit erosive radiographic features indistinguishable from RA, which underscores the necessity for more nuanced assessment methodologies when evaluating conditions with overlapping phenotypic expressions.

Clinical immunological studies demonstrate RF and anti-CCP antibodies are widely present in RA-SS patient sera. The production of these antibodies is closely associated with protein citrullination and plays a key role in disease pathogenesis and articular destruction progression. RF has been used as an RA marker for over five decades and remains the most common diagnostic indicator. The literature reports RF positivity in 70-90% of RA cases and 36-74% of SS patients (32, 33). In contrast, anti-CCP, secreted by B lymphocytes, demonstrates positivity in early RA stages and shows high sensitivity and specificity for RA patients, serving as a highly specific marker for early diagnosis and progression prediction (34).

The SHAP analysis revealed anti-CCP level, RF level, and erosive joint count as critical factors for distinguishing RA-SS. These results are highly consistent with research by Yang et al. (35) establishing arthritis, RF, and anti-CCP as independent risk factors for RA-SS overlap. A retrospective analysis of 355 patients found that RF and anti-CCP levels, anti-Ro/SS-A positivity, and renal involvement can effectively differentiate high-risk RA-SS populations, consistent with the immunological marker profile identified in this study (27). Anti-Ro60 antibodies frequently occur in SS but may participate in more complex immunopathological processes in RA-SS patients. Studies have indicated that anti-Ro60 positive RA patients exhibited stronger inflammatory responses and more severe joint destruction, possibly due to Ro60-mediated RNA metabolic abnormalities enhancing inflammatory cascades (36, 37). Methodologically, their study primarily utilized conventional clinical serology and systemic manifestations for analysis (27), whereas the present investigation incorporated more refined joint damage assessment parameters. As a core pathological feature of RA, quantitative assessment of joint erosion may provide more direct evidence for RA-SS differential diagnosis.

Notably, previous studies have provided valuable clinical evidence but relied primarily on LR for risk factor analysis (27, 35). This approach may insufficiently capture complex multidimensional features and non-linear relationships in autoimmune disorders. Machine learning algorithms have become increasingly used in medical applications due to their capacity to analyze complex data structures and recognize latent patterns. This study evaluated four distinct machine learning algorithms for RA-SS/SS-PA differential diagnosis. Among the evaluated algorithms, the random forest demonstrated superior performance over both logistic regression and other machine learning methods. The ensemble nature of random forest, combined with its ability to capture non-linear relationships and complex feature interactions, proved particularly effective for distinguishing between these clinically overlapping autoimmune conditions. The algorithm’s performance was further enhanced by the focused feature set obtained through LASSO regression preprocessing. While deep learning approaches continue to advance rheumatological diagnostics, our study design prioritized traditional machine learning methodologies based on the specific characteristics of our clinical dataset. The mixed nature of our variables, comprising both categorical and continuous clinical parameters, aligned well with the strengths of ensemble-based algorithms. Furthermore, the emphasis on clinical interpretability in differential diagnosis guided our selection toward methods that provide transparent decision pathways for practitioners (38).

This study has several limitations. First, the retrospective design introduces selection bias that may affect result generalizability. Second, this was a single-center study, which may limit external validity across different populations and clinical settings. Third, while our sample size of 241 patients is adequate for binary classification studies, it remains relatively limited for comprehensive machine learning modeling, particularly for deep learning approaches, which may limit statistical power and affect the precision of performance estimates. Fourth, our validation strategy employed a single train-test split approach rather than cross-validation. While this approach avoids data leakage and provides unbiased performance estimates, it uses less data for training and provides performance estimates based on one specific data partition, which may be less robust than estimates derived from multiple validation rounds. Furthermore, the radiographic assessment was restricted to three parameters (erosive joint count, hand joint involvement proportion, and symmetric arthritis distribution) without incorporating comprehensive structural damage indices or advanced imaging modalities, potentially limiting complete characterization of articular pathology. Future prospective multicenter studies with larger cohorts and advanced imaging are needed to improve diagnostic accuracy. Expanded datasets would also enable exploration of deep learning methodologies, which may offer enhanced pattern recognition capabilities for complex autoimmune disease classification tasks.

## Conclusion

In conclusion, this study demonstrates the substantial clinical utility of the random forest algorithm in differentiating RA-SS from SS-PA, with the identification of key discriminatory parameters including anti-CCP and RF levels, erosive joint count, anti-SSA/Ro60 positivity, and CRP value. These findings enhance the diagnostic criteria available to clinicians, potentially facilitating earlier detection and therapeutic intervention in RA-SS cases, with subsequent implications for improved patient outcomes.

## Data Availability

The original contributions presented in the study are included in the article/**Supplementary Material**. Further inquiries can be directed to the corresponding author.
