# Supplementary material for: Machine learning differentiation of rheumatoid arthritis-Sjögren’s syndrome overlap from Sjögren’s syndrome with polyarthritis
Source: Front Immunol. 2025 Jul 8;16:1614631. doi: 10.3389/fimmu.2025.1614631 (PMC12280366; doi:10.3389/fimmu.2025.1614631)
Supplement: Supplementary file 1 [file DataSheet1.docx]

import pandas as pd

import numpy as np

import tkinter as tk

from tkinter import filedialog

import matplotlib.pyplot as plt

import seaborn as sns

from sklearn.model_selection import GridSearchCV, cross_val_score, StratifiedKFold

from sklearn.preprocessing import StandardScaler, LabelEncoder

from sklearn.linear_model import LogisticRegression, LassoCV

from sklearn.svm import SVC

from sklearn.ensemble import RandomForestClassifier

from sklearn.metrics import classification_report, confusion_matrix, roc_auc_score, roc_curve

import joblib

import os

from datetime import datetime

import warnings

warnings.filterwarnings('ignore')

# Try to import XGBoost with compatibility check

try:

import xgboost as xgb

XGB_AVAILABLE = True

print("✓ XGBoost imported successfully")

except ImportError:

XGB_AVAILABLE = False

print("✗ XGBoost not available. Install with: pip install xgboost")

except Exception as e:

XGB_AVAILABLE = False

print(f"✗ XGBoost import error: {e}")

# Version compatibility check

try:

import sklearn

print(f"ℹ Scikit-learn version: {sklearn.__version__}")

if XGB_AVAILABLE:

print(f"ℹ XGBoost version: {xgb.__version__}")

except:

pass

class MLModelTrainer:

def __init__(self):

self.data = None

self.X = None

self.y = None

self.X_scaled = None

self.selected_features = None

self.scaler = StandardScaler()

self.models = {}

self.best_models = {}

self.cv_scores = {}

def load_data(self):

print("Please select your Excel file...")

root = tk.Tk()

root.withdraw() # Hide the main window

file_path = filedialog.askopenfilename(

title="Select Excel file",

filetypes=[("Excel files", "*.xlsx *.xls")]

)

if not file_path:

print("No file selected!")

return False

try:

self.data = pd.read_excel(file_path)

print(f"Data loaded successfully! Shape: {self.data.shape}")

print(f"Columns: {list(self.data.columns)}")

# Check if Group column exists

if 'Group' not in self.data.columns:

print("Error: 'Group' column not found in the data!")

return False

# Separate features and target

self.y = self.data['Group'].values

self.X = self.data.drop('Group', axis=1)

print(f"Target distribution: {pd.Series(self.y).value_counts().to_dict()}")

print(f"Number of features: {self.X.shape[1]}")

return True

except Exception as e:

print(f"Error loading data: {str(e)}")

return False

def preprocess_data(self):

print("\nPreprocessing data...")

# Handle missing values

numeric_columns = self.X.select_dtypes(include=[np.number]).columns

categorical_columns = self.X.select_dtypes(include=['object']).columns

# Fill missing values for numeric columns with median

for col in numeric_columns:

if self.X[col].isnull().sum() > 0:

self.X[col].fillna(self.X[col].median(), inplace=True)

# Encode categorical variables

label_encoders = {}

for col in categorical_columns:

if self.X[col].isnull().sum() > 0:

self.X[col].fillna(self.X[col].mode()[0], inplace=True)

le = LabelEncoder()

self.X[col] = le.fit_transform(self.X[col].astype(str))

label_encoders[col] = le

# Standardize features (z-score normalization: mean=0, std=1)

self.X_scaled = self.scaler.fit_transform(self.X)

self.X_scaled = pd.DataFrame(self.X_scaled, columns=self.X.columns)

print("Data preprocessing completed!")

print(f"Final feature shape: {self.X_scaled.shape}")

def lasso_feature_selection(self, cv_folds=10):

print(f"\nPerforming LASSO feature selection with {cv_folds}-fold cross-validation...")

# LASSO with cross-validation to find optimal lambda

lasso_cv = LassoCV(cv=cv_folds, random_state=42, max_iter=2000)

lasso_cv.fit(self.X_scaled, self.y)

# Get selected features (non-zero coefficients)

selected_mask = lasso_cv.coef_ != 0

self.selected_features = self.X_scaled.columns[selected_mask].tolist()

print(f"Optimal alpha (lambda): {lasso_cv.alpha_:.6f}")

print(f"Selected {len(self.selected_features)} features out of {self.X_scaled.shape[1]}")

print(f"Selected features: {self.selected_features}")

# Update X_scaled to include only selected features

self.X_scaled = self.X_scaled[self.selected_features]

def setup_models(self):

print("\nSetting up machine learning models...")

print("Hyperparameter configurations:")

print("- LR: L2 regularization, C logarithmically spaced from 0.001 to 10")

print("- SVM: RBF kernel, γ (0.001-0.1), C (1-100), tolerance 0.0001")

print("- RF: Trees (100-500), max depth (4-8), min samples split (2-10), bootstrap sampling")

print("- XGBoost: Learning rate (0.01-0.2), trees (100-300), max depth (3-6), ratios 0.8")

# Logistic Regression with L2 regularization

# Strength parameters logarithmically spaced from 0.001 to 10

lr_param_grid = {

'C': np.logspace(-3, 1, 15) # 0.001 to 10, more granular search

}

# Support Vector Machine with RBF kernel

# Gamma logarithmically spaced from 0.001 to 0.1, C from 1 to 100, tolerance 0.0001

svm_param_grid = {

'gamma': np.logspace(-3, -1, 10), # 0.001 to 0.1

'C': np.logspace(0, 2, 15), # 1 to 100

'kernel': ['rbf'], # RBF kernel

'tol': [0.0001] # tolerance of 0.0001

}

# Random Forest with bootstrap sampling

# Tree numbers (100-500), max depth (4-8), min samples for split (2-10)

rf_param_grid = {

'n_estimators': [100, 200, 300, 400, 500],

'max_depth': [4, 5, 6, 7, 8],

'min_samples_split': [2, 4, 6, 8, 10],

'bootstrap': [True] # Bootstrap sampling enabled

}

# XGBoost parameters

# Learning rates (0.01-0.2), trees (100-300), max depth (3-6), subsample and feature ratios at 0.8

xgb_param_grid = {

'learning_rate': [0.01, 0.05, 0.1, 0.15, 0.2],

'n_estimators': [100, 150, 200, 250, 300],

'max_depth': [3, 4, 5, 6],

'subsample': [0.8],

'colsample_bytree': [0.8]

}

# Test XGBoost compatibility

xgb_works = False

if XGB_AVAILABLE:

try:

# Try XGBoost with sklearn interface

xgb_model = xgb.XGBClassifier(

random_state=42,

eval_metric='logloss',

verbosity=0, # Reduce output

use_label_encoder=False

)

# Test if XGBoost works with sklearn

from sklearn.model_selection import cross_val_score

from sklearn.datasets import make_classification

X_test, y_test = make_classification(n_samples=100, n_features=10, random_state=42)

cross_val_score(xgb_model, X_test, y_test, cv=2) # Quick test

xgb_works = True

print("✓ XGBoost compatibility test passed")

except Exception as e:

print(f"✗ XGBoost sklearn interface error: {str(e)[:100]}...")

print("Skipping XGBoost due to compatibility issues")

xgb_works = False

else:

print("ℹ XGBoost not available, skipping")

self.models = {

'Logistic Regression': {

'model': LogisticRegression(penalty='l2', random_state=42, max_iter=1000),

'params': lr_param_grid

},

'SVM': {

'model': SVC(probability=True, random_state=42), # tolerance will be set by param grid

'params': svm_param_grid

},

'Random Forest': {

'model': RandomForestClassifier(random_state=42), # bootstrap will be set by param grid

'params': rf_param_grid

}

}

# Only add XGBoost if it works

if xgb_works:

self.models['XGBoost'] = {

'model': xgb_model,

'params': xgb_param_grid

}

print("✓ XGBoost added to model list")

else:

print("ℹ Continuing with 3 models: LR, SVM, Random Forest")

print(f"Models setup completed! Total models: {len(self.models)}")

print(f"Model names: {list(self.models.keys())}")

def train_models(self, cv_folds=10):

print(f"\nTraining models with {cv_folds}-fold cross-validation...")

print(f"Models to train: {list(self.models.keys())}")

# Setup stratified k-fold cross-validation

skf = StratifiedKFold(n_splits=cv_folds, shuffle=True, random_state=42)

for name, model_info in self.models.items():

print(f"\nTraining {name}...")

try:

# Grid search with cross-validation

grid_search = GridSearchCV(

estimator=model_info['model'],

param_grid=model_info['params'],

cv=skf,

scoring='roc_auc',

n_jobs=-1,

verbose=1

)

grid_search.fit(self.X_scaled, self.y)

# Store best model and scores

self.best_models[name] = grid_search.best_estimator_

self.cv_scores[name] = grid_search.best_score_

print(f"✓ {name} training completed successfully")

print(f"Best parameters for {name}: {grid_search.best_params_}")

print(f"Best CV AUC score for {name}: {grid_search.best_score_:.4f}")

except Exception as e:

print(f"✗ Error training {name}: {str(e)}")

print(f"Skipping {name} due to training error")

continue

def evaluate_models(self, cv_folds=10):

print(f"\nEvaluating models with {cv_folds}-fold cross-validation...")

skf = StratifiedKFold(n_splits=cv_folds, shuffle=True, random_state=42)

results = {}

for name, model in self.best_models.items():

# Cross-validation scores

cv_scores = cross_val_score(model, self.X_scaled, self.y, cv=skf, scoring='roc_auc')

# Fit model for predictions

model.fit(self.X_scaled, self.y)

y_pred = model.predict(self.X_scaled)

y_pred_proba = model.predict_proba(self.X_scaled)[:, 1]

# Calculate metrics

auc_score = roc_auc_score(self.y, y_pred_proba)

results[name] = {

'CV_AUC_mean': cv_scores.mean(),

'CV_AUC_std': cv_scores.std(),

'AUC': auc_score,

'predictions': y_pred,

'probabilities': y_pred_proba

}

print(f"\n{name} Results:")

print(f"Cross-validation AUC: {cv_scores.mean():.4f} ± {cv_scores.std():.4f}")

print(f"AUC Score: {auc_score:.4f}")

print("\nClassification Report:")

print(classification_report(self.y, y_pred))

return results

def save_models(self, results, save_path=None):

"""Save all trained models and related components"""

if save_path is None:

save_path = f"trained_models_{datetime.now().strftime('%Y%m%d_%H%M%S')}"

# Create directory if it doesn't exist

os.makedirs(save_path, exist_ok=True)

# Save all individual models

for name, model in self.best_models.items():

model_filename = os.path.join(save_path, f"{name.lower().replace(' ', '_')}_model.pkl")

joblib.dump(model, model_filename)

print(f"Saved {name} model to: {model_filename}")

# Save scaler

scaler_path = os.path.join(save_path, "scaler.pkl")

joblib.dump(self.scaler, scaler_path)

# Save selected features

features_path = os.path.join(save_path, "selected_features.pkl")

joblib.dump(self.selected_features, features_path)

# Save model performance results

performance_data = []

for name, result in results.items():

performance_data.append({

'Model': name,

'CV_AUC_mean': result['CV_AUC_mean'],

'CV_AUC_std': result['CV_AUC_std'],

'AUC': result['AUC']

})

performance_df = pd.DataFrame(performance_data)

performance_path = os.path.join(save_path, "model_performance.csv")

performance_df.to_csv(performance_path, index=False)

print(f"\nAll models and components saved to: {save_path}")

return save_path

def run_complete_analysis(self):

print("Starting Complete ML Analysis Pipeline...")

print("=" * 50)

# Load data

if not self.load_data():

return None

# Preprocess data

self.preprocess_data()

# Feature selection

self.lasso_feature_selection()

# Setup models

self.setup_models()

# Check if we have any models to train

if not self.models:

print("❌ No models available for training!")

print("This might be due to XGBoost compatibility issues.")

print("Please check your sklearn and xgboost versions:")

print(" pip install --upgrade scikit-learn xgboost")

return None

# Train models

self.train_models()

# Check if any models were successfully trained

if not self.best_models:

print("❌ No models were successfully trained!")

return None

# Evaluate models

results = self.evaluate_models()

print(f"\n✅ Analysis completed successfully!")

print(f"Successfully trained {len(self.best_models)} models: {list(self.best_models.keys())}")

return results

# Main execution

if __name__ == "__main__":

print("ML Model Trainer for RA-SS vs SS-PA Classification")

print("=" * 60)

# Check for potential compatibility issues

if not XGB_AVAILABLE:

print("⚠️ Warning: XGBoost not available")

print(" To install: pip install xgboost")

print(" Training will continue with 3 models (LR, SVM, RF)")

print("=" * 60)

# Direct training mode

trainer = MLModelTrainer()

# Run complete model training

results = trainer.run_complete_analysis()

# Save models and results

if results:

print("\nWould you like to save the trained models? (y/n)")

save_choice = input().lower()

if save_choice == 'y':

# Ask for custom save path

print("Enter a custom folder name (or press Enter for default timestamped folder):")

custom_path = input().strip()

if custom_path:

save_path = custom_path

else:

save_path = None # Will use default timestamped folder

# Save all models and components

saved_path = trainer.save_models(results, save_path)

print(f"\n{'='*60}")

print("ALL MODELS SAVED SUCCESSFULLY!")

print(f"{'='*60}")

print(f"Location: {saved_path}")

print("Files saved:")

# List only the models that were actually trained

for model_name in trainer.best_models.keys():

model_filename = f"{model_name.lower().replace(' ', '_')}_model.pkl"

print(f" - {model_name} model ({model_filename})")

print(" - Data scaler (scaler.pkl)")

print(" - Selected features (selected_features.pkl)")

print(" - Model performance (model_performance.csv)")

print(f"{'='*60}")

else:

print("Models not saved. Training results available in memory.")

else:

print("❌ Training failed. Please check your data and try again.")

print("\n🔧 Troubleshooting tips:")

print("1. Ensure your Excel file has a 'Group' column")

print("2. Check for sufficient data samples")

print("3. Update packages: pip install --upgrade scikit-learn xgboost pandas")

if not XGB_AVAILABLE:

print("4. Install XGBoost: pip install xgboost")
